# Frailty and Oral Health: Associations with Candidiasis, Prosthesis Use, and Hyposalivation

**DOI:** 10.3390/geriatrics10050116

**Published:** 2025-08-26

**Authors:** Renata Centanaro-Vera, Fuad Huamán-Garaicoa, Sofia Cuadrado-Rios, Marcelo Armijos, Ludwig Álvarez-Córdova, Karla Cruz-Moreira

**Affiliations:** 1 Dentistry Degree Program, Catholic University of Santiago de Guayaquil, Guayaquil 090615, Guayas, Ecuador; renatacvera14@gmail.com; 2 Department of Pathology, Instituto Oncológico Nacional SOLCA Guayaquil, Guayaquil 090615, Guayas, Ecuador; fuadhuamangaraicoa@gmail.com; 3 Department of Research, School of Dentistry, Catholic University of Santiago de Guayaquil, Guayaquil 090615, Guayas, Ecuador; 4 School of dentistry, Universidad de Especialidades Espíritu Santo, Samborondón 092301, Guayas, Ecuador; fernando.armijos@cu.ucsg.edu.ec; 5 School of Medicine, Universidad de las Américas (UDLA), Quito 170513, Pichincha, Ecuador; ludwig.alvarez@udla.edu.ec; 6 Instituto de Investigación e Innovación en Salud Integral, Catholic University of Santiago de Guayaquil, Guayaquil 090615, Guayas, Ecuador; karla.cruz@cu.ucsg.edu.ec; 7 Department of Oral Medicine, Catholic University of Santiago de Guayaquil, Guayaquil 090615, Guayas, Ecuador; 8 Oral Medicine Department, Úraga Dermatología y Estética, Guayaquil 090615, Guayas, Ecuador

**Keywords:** frailty, oral candidiasis, hyposalivation, dental prosthesis

## Abstract

**Background/Objectives**: Frailty syndrome favors the deterioration of health; therefore, identifying associated factors is essential for establishing preventive measures. Oral candidiasis is a factor that may be related to the onset of frailty. Our objective was to evaluate the association between frailty and oral candidiasis in institutionalized participants. **Methods**: We conducted a cross-sectional study involving 589 institutionalized individuals aged 65 years or older. A diagnosis of candidiasis was established clinically and with a microbiological study (potassium hydroxide (KOH) test and culture for candidiasis). Assessments of salivary flow and the use of dental prostheses were also performed. Frailty was classified according to Fried’s phenotype criteria. **Results**: Frailty and prefrailty were found in 28.9% and 66.7% of the participants, respectively. Oral candidiasis was diagnosed in 39.05% of cases and was more frequent among individuals with dental prostheses (49.13%) and hyposalivation (47.54%). **Conclusions**: Our findings suggest that frailty in institutionalized older adults is associated with the presence of subprosthetic stomatitis associated with candidiasis and hyposalivation, indicating the need for integrated oral health strategies in geriatric care.

## 1. Introduction

Aging is a biological and natural process that is part of the life cycle and is characterized by being progressive and irreversible, causing the deterioration of the physical and psychological functions of older people, limiting their functions, and even increasing mortality [1,2,3]. The World Health Organization (WHO) warns that the pace of population aging is currently much faster than that in the past: between 2015 and 2050, the proportion of the world’s population over 60 years of age is projected to nearly double from 12% to 22% [4]. This demographic shift poses significant challenges for health systems, particularly in addressing chronic diseases and geriatric syndromes.

One of the main complications associated with aging is frailty syndrome, which is characterized by a decrease in physiological reserves, increasing susceptibility to endogenous and exogenous stressors [5,6]. This syndrome usually appears in adults over 65 years of age and has a worldwide prevalence between 3.9% and 51.4%, with a higher prevalence in women [7,8]. The main complications include increased vulnerability to falls, increased susceptibility to different diseases and changes in physical function. On the other hand, frailty can also present psychological problems due to changes caused by disability or dependence [9,10,11].

Decreased physiological function remains the primary symptom and is the primary trigger for declining health in older adults [12,13,14]. For this reason, identifying all associated factors is fundamental for establishing preventive measures.

One of the factors that has been associated with frailty is deterioration of the oral cavity, and various associated problems have been identified [15]. Oral candidiasis is an infection caused by fungi of the genus *Candida*, whose risk of occurrence in older adults is increased by poor hygiene, the use of dental prostheses, reduced salivary secretion and depression of the immune system, etc. This infection has a prevalence of 9.4% in older adults, with women being the most affected [16]. There are several species involved in this disease, such as *Candida albicans*, *C. glabrata*, *C. krusei*, and *C. guilliermondii*, among others, which can generate different lesions. Oral candidiasis presents in several clinical forms; acute variants include acute pseudomembranous candidiasis, commonly known as thrush, and acute atrophic candidiasis, which is often linked to antibiotic use or other iatrogenic factors [17]. On the other hand, chronic forms comprise chronic atrophic candidiasis—the most frequent in denture wearers—and chronic hyperplastic candidiasis, a non-wipeable white lesion that is frequently associated with tobacco use [17]. Another condition that commonly affects older adults is subprosthetic stomatitis, which may result from multiple causes, including *Candida* infection, hyposalivation and ill-fitting dentures [17]. This entity manifests as the diffuse inflammation of the maxillary denture-bearing mucosa, and may be accompanied by angular cheilitis in 15–65% of cases [18]. Symptoms of burning, pain and dysphagia cause changes in the nutritional status of the host, increasing the risk of fragility [19,20,21].

The objective of this cross-sectional study was to determine the association between frailty and oral candidiasis in institutionalized older adults. Understanding this association may help develop targeted oral health interventions that could mitigate frailty progression in institutionalized populations.

## 2. Materials and Methods

The following is a descriptive, cross-sectional study that was approved by the Ethics Committee of Hospital Clínica Kennedy (approval no. HCK-CEISH-19-0036) and conformed to the Declaration of Helsinki on human research. It was carried out in 10 residences in the city of Guayaquil, Ecuador (Luis Plaza Dañin Home, San José Home Asylum, Vida Plena Gerontological Center, Corazón de Jesús Asylum/Guayaquil Charity Board, La Esperanza #2 Home, Sofia Ratinoff Gerontological Center, Iglesia Elevation Municipal Gerontological Center, Orchids Municipal Gerontological Center, Dr. Arsenio de la Torre Marcillo Municipal Gerontological Center and Senior Citizen Club). All the older adults who met the following inclusion criteria were examined: (1) agreed to participate in the study and provided informed consent, (2) were temporary or transient residents of the nursing homes, (3) were aged ≥65 years, and (4) did not present with cognitive impairment (Mini-Mental State Examination (MMSE) score > 23/30). Those with (1) a history of stroke or Parkinson’s or Alzheimer’s disease or (2) an inability to walk were excluded. Notably, the data were collected from January 2018 to December 2019. The intraoral examination was performed by three dentists, and the frailty assessment was performed by three nutritionists. The team members participated in calibration sessions before the start of the study. Ten older adults were examined for the calculation of inter-examiner reliability. Interclass correlation coefficients ranged from 0.97 to 0.99 among both the dentists and dietitian nutritionists.

### 2.1. Assessment of Fragility

The presence of frailty was established through Fried’s phenotype [22] which evaluates 5 components:

1. Exhaustion: two questions from the Center for Epidemiological Studies Depression Scale (CESD-R) were used: “I felt that everything I did was an effort last week” and “I could not keep up with my routine last week”. Frequent or “always” responses to either question indicated positivity for burnout.
2. Low physical activity: participants were asked about their physical activities on the basis of the Minnesota Leisure Activities Questionnaire (MLTAQ). To calculate the score, the kilocalories expended per week were recorded, which were positive if men expended <383 kcal and women expended <270 kcal.
3. Slowness: a stopwatch was used to measure the 4.6 m walking time. It was considered positive in men (length/time) if it was ≤173 cm/≥7 s or >173 cm/≥6 s, and in women if it was ≤159 cm/≥7 s or >159 cm/≥6 s.
4. Weakness: a dynamometer (Jamar^TM^ hydraulic hand dynamometer 50/30 J1, Chicago, IL, USA) was used to measure grip strength in the dominant hand. Strength was measured and adjusted for sex and body mass index (BMI) and recorded in kilograms. The criteria outlined by Fried et al. [22] were used.
5. Weight loss: loss ≥ 10 lb (4.5 kg), unintentional in the past year compared with the previous year, was considered positive [22].

Participants with 3 or more components present were considered “fragile”, those with 2 or 1 component were “prefragile”, and in the absence of such components, the participants were categorized as “robust”.

### 2.2. Intraoral Evaluation

The intraoral examination consisted of evaluating the soft tissues for clinical lesions of candidiasis (pseudomembranes, erythema, atrophy, papules, or hyperplasia). If signs were found, swabbing was performed by rubbing the swab against the lesion, which was then placed in the transport medium for subsequent microbiological analysis to determine the presence of bacteria and fungi. Fungal evaluation was conducted using potassium hydroxide (KOH) preparation and culture for *Candida* species. All patients presented with common oral cavity microorganisms; however, no pathogenic bacteria were detected. Patients with a positive mycological study for *Candida* species were assigned to the candidiasis group and further classified according to the clinical type. Those with negative results continued to be evaluated to establish an accurate diagnosis and appropriate treatment; nevertheless, they were categorized within the non-candidiasis group. The use of dental prostheses and manifestations of subprosthetic stomatitis were subsequently recorded. Saliva samples were collected between 7:00 and 8:00 a.m., prior to tooth brushing, for sialometry analysis (unstimulated salivary flow).

### 2.3. Other Variables

Data on sociodemographic aspects (sex, age), smoking, alcohol consumption and chronic diseases (hypertension, diabetes mellitus, heart disease, osteoarticular diseases, osteoporosis, etc.) were collected via a standardized questionnaire.

### 2.4. Statistical Analysis

A descriptive analysis of the sample was performed, and the means and standard deviations of the continuous quantitative variables were calculated. For qualitative variables, the distributions of absolute and relative frequencies were calculated. The chi-square test was performed to evaluate the associations between sociodemographic characteristics and the presence of frailty. Statistical analyses were performed via STATA 15.0 software (Stata Corp. LP, College Station, TX, USA).

## 3. Results

### 3.1. Sociodemographic Characteristics

Of the 942 older adults housed in private and public residences in the city of Guayaquil, only 589 met the inclusion criteria. The minimum age of these patients was 65 years, the maximum age was 100, and the mean age was 72.2 years. More than half of the participants (65%) were female. The age group was classified as follows:

- Aged 65—74 years (“early old age”): 55.7%;
- Aged 75—79 years (“intermediate old age”): 13.2%;
- Aged ≥80 years (“advanced old age”): 31.1%.

Only 28.0% were married. The most prevalent systemic disease was arterial hypertension (23.6%). In terms of toxic habits,

1. A total of 22.8% smoked tobacco.
2. A total of 32.1% drank alcohol.

On the other hand, 66.9% of the participants used some type of removable dental prosthesis.

### 3.2. Frailty Profile

In relation to frailty,

- A total of 4.4% of the total number of participants were considered robust;
- A total of 66.7% met the criteria for prefrailty;
- A total of 28.9% were categorized as frail.

By sex, women presented a higher prevalence of this syndrome compared with men. However, the difference did not reach statistical significance (*p* value = 0.171).

- Robust: women 3.7%, men 5.8%;
- Prefragile: women 69.2%, men 62.1%;
- Fragile: women 27.1%, men 32.0%.

No significant association was found between the presence of frailty and prefrailty with systemic diseases; however, it should be noted that more than 50% of the participants with hypertension and diabetes mellitus were considered prefrail. The highest prevalence of frailty was in participants with concomitant hypertension and diabetes (Table 1).

### 3.3. Oral Candidiasis

Clinically, manifestations suggestive of oral candidiasis were observed in 39.05% of cases. Culture testing was performed in these patients, with only 20.71% yielding positive results, thereby confirming the diagnosis. Women being the most affected (66.39%). The most prevalent clinical forms were:

- Hyperplastic candidiasis (46.72%);
- Erythematous candidiasis (29.51%).

Regarding the positive diagnosis of candidiasis and its anatomical localization within the oral cavity, three specific sites were identified, along with their most frequent clinical presentations (Table 2):

- Tongue: hyperplastic candidiasis (68.7%) and pseudomembranous candidiasis (18.1%);
- Palate: erythematous candidiasis (86.8%);
- Buccal mucosa: atrophic candidiasis (100%).
- When sociodemographic aspects were related to this infection, erythematous candidiasis was more common in females (43.2%), whereas hyperplastic candidiasis was more common in males (53.7%).

In addition, 103 (84.42%) of the participants who presented a positive culture for Candidiasis (*n* = 122) used some type of dental prosthesis, and a relationship was found between the use of prostheses and the presence of erythematous candidiasis (35.0%, *p* = 0.001) and hyperplastic candidiasis (45.6%, *p* = 0.001) (Table 3).

### 3.4. Subprosthetic Stomatitis Associated with Candidiasis and Hyposalivation

Subprosthetic stomatitis associated with Candidiasis was found in 103 patients, with women being more affected (68.9%). For its part, hyposalivation is a factor that contributes to the appearance of oral candidiasis and subprosthetic stomatitis, and it was found that 50.5% presented this condition, which was also more prevalent in women (53.5%). Notably, a statistically significant association was observed between

- prefrailty and frailty, with the presence of subprosthetic stomatitis associated with candidiasis and hyposalivation (55.8% and 44.2%, respectively; *p* = 0.004). (Table 4).

### 3.5. Candidiasis Species

The most prevalent species type was *C. albicans* at 70.7%; however, no significant association was found between frailty and any of the species of candidiasis.

## 4. Discussion 

As the population ages, the prevalence of frailty increases; therefore, this public health problem cannot be ignored. In China, approximately 10% of older people aged 60 years suffer from frailty, and 15% of those aged 75–84 years suffer from frailty, whereas 25% of older adults aged 85 years suffer from frailty [23,24,25]. Thus, it can be inferred that approximately 50% of older adults present frailty and prefrailty [26].

Based on the results of this study, the prevalence of prefrailty (66.7%) and frailty (28.9%) was high, which was expected since frailty is a predisposing factor for institutionalization. These results agree with the meta-analysis by Veronese et al. [27] (2021), who evaluated frailty in different settings (ambulatory senile populations, nursing homes, and hospitalized older people) and reported a prevalence of frailty of 26.8%, with frailty being higher in institutionalized older adults. However, in our study, the prevalence of prefrailty was greater than that in this meta-analysis, where a prevalence of prefrailty of 36.4% was reported to be greater in hospitalized older adults (39.3%) and lower in nursing homes (20%). This possible discrepancy could be due to the method used to measure frailty, since this study used the Fried phenotype while that of Veronese et al. [27] used the multidimensional prognostic index (MPI). On the other hand, women were the most affected by this syndrome, with only 3.7% considered robust, which coincides with the literature, where it has been observed that women remain more susceptible [28,29].

Around the world, millions of older adults suffer from chronic diseases; for example, in China, the prevalence is 43.6%, with hypertension and diabetes being the most common [30]. Chronic diseases shorten life expectancy and favor the onset of other diseases, such as frailty [26]. In this study, the highest prevalence of frailty was found in older adults with concomitant hypertension and diabetes (39.3%), although no statistically significant associations were found with these or other systemic diseases. Vetrano et al. [31] (2018) reported that the results of their systematic review and meta-analysis were contradictory regarding the cross-sectional association between frailty and hypertension. Among the 23 studies evaluated, 13 reported a significantly greater prevalence of frailty in hypertensive participants, whereas 10 reported no significant association. On the other hand, although we did not find a statistically significant association in our study, it is worth highlighting that more than 50% of the participants considered prefrail presented hypertension and diabetes mellitus, which agrees with some authors who have indicated that the prevalence of hypertension and diabetes is greater when frailty is present [31,32].

Oral pathologies, such as candidiasis, which is an infection caused by a yeast-like fungus of the genus *Candida*, are among the main problems affecting older adults [33]. This microorganism is normally found in the oral cavity of 30 to 60% of the population [34]. Although it is commensal, various local and systemic conditions can enhance its transformation into an opportunist. These conditions include alterations in salivary secretion caused by polypharmacy, the use of dental prostheses, immunological and nutritional deficiencies, and even chronic diseases such as diabetes mellitus [35]. This study revealed that 20.71% of institutionalized older adults were diagnosed with oral candidiasis, with a higher prevalence in women (66.39%). Radwan-Oczko et al. [16] (2022) reported that women (10.4%) are more susceptible to oral candidiasis than men (6.6%), reporting a prevalence of this pathology of 9.4%, possibly because the participants in that study were slightly younger (60–93 years), with a mean age of 69 years. On the other hand, the participants of Radwan-Oczko et al. [16] were outpatients, whereas our participants, who were institutionalized, presented more limitations in their oral health care.

The clinical forms of oral candidiasis are diverse and include acute pseudomembranous candidiasis, acute erythematous candidiasis (also called acute atrophic candidiasis), chronic hyperplastic candidiasis, chronic atrophic candidiasis, medial rhomboid glossitis, and angular cheilitis [36]. To date, no studies have evaluated the prevalence of the clinical forms of candidiasis found in institutionalized older adults. However, this study revealed that the most common clinical form in older adults living in nursing homes was chronic hyperplastic candidiasis (46.72%), followed by erythematous candidiasis (29.51%). Several authors have indicated that tobacco consumption increases the incidence of chronic hyperplastic candidiasis, as well as the presence of rough surfaces within the oral cavity; although it is usually painless, it is common to observe it on the lateral edges of the tongue because they are areas of friction [17,36]. Erythematous candidiasis, on the other hand, is usually painful and is clinically observed as burning erythema. Chronic atrophic candidiasis is asymptomatic and is commonly found in patients with dentures [37]. Acute pseudomembranous candidiasis usually involves white plaques that detach when scraped, which are asymptomatic and are associated with salivary gland hypofunction, xerostomia, and immunosuppression, among other conditions [38,39].

The presence of dental prostheses was an important variable since there was a correlation between their use and the diagnosis of acute erythematous and chronic hyperplastic candidiasis in 35.0% and 45.6%, respectively, which agrees with the findings of Cueto et al. [40] (2013), who reported a statistically significant association between the use of prostheses and the presence of oral candidiasis. However, our findings differ from those of other authors, who reported that chronic atrophic candidiasis is the most common type of lesion associated with prosthesis use [17], and that owing to this association, it is also known as subprosthetic stomatitis associated with Candidiasis [17]. On the other hand, Adam and Kimmie-Dhansay [41] (2021) reported that the site of greatest susceptibility was the palate (40.2%), which differs from our findings, where the greatest involvement was on the dorsum of the tongue. This difference is because we evaluated the types of candidiasis by their clinical manifestations and in any location, whereas Adam evaluated the occurrence of candidiasis only in relation to the location of the prosthesis.

Subprosthetic stomatitis is an inflammatory lesion frequently associated with the use of a prosthesis, and its etiology is unknown; however, *Candida* infection is usually related, in addition to other factors, such as poor hygiene and xerostomia [19]. Clinically, it can be observed as areas of erythema, atrophy, and papules on the mucosa supporting the prosthesis [42]. In our study (*n* = 103), 84.42% of the participants presented subprosthetic stomatitis associated with Candidiasis with greater effects on women, which coincides with the findings of another author, who reported (*n* = 102; 25.76%) a relationship between stomatitis and the use of prostheses and greater vulnerability in females (75%) [41]. On the other hand, subprosthetic stomatitis can affect the oral mucosa and has the potential to compromise essential functions such as mastication, maintenance of oral hygiene, and overall oral comfort. These impairments may contribute to oral hypofunction through a progressive decline in oral functional capacity, which, in turn, may increase the risk of developing frailty [10].

Another variable that was analyzed was hyposalivation, which is characterized by a decrease in salivary flow, which results in oral dryness and favors *Candida* colonization. Among our participants, hyposalivation was found in 47.54%, with more than half of them being women (66.43%). These data are like those of Buranarom et al. [43] (2020), who also reported a higher prevalence of hyposalivation in women, in addition to finding a statistically significant association between hyposalivation (41.5%) with systemic diseases and the use of dental prostheses. The same author reported that hyposalivation is positively associated with the diagnosis of oral candidiasis (*p* = 0.010) and that its occurrence increases with the use of dental prostheses (*p* = 0.017), which agrees with our findings [43].

Importantly, a statistically significant association was found between fragility, subprosthetic stomatitis associated with Candidiasis and hyposalivation (44.2%); however, we did not find studies that analyzed the same association for discussion. Finally, regarding *Candida* species, one study reported that the most prevalent strain was *C. albicans* (73.40%), followed by *C. glabrata* (26.60%), while both were detected in 13.3% of participants [14]. In our study, *C. albicans* was the most common species (70.7%) among fragile and prefragile participants, although a statistically significant relationship was not detected.

## 5. Conclusions

The prevalence of frailty and prefrailty in institutionalized older adults was high, and a statistically significant association was found with the presence of subprosthetic stomatitis associated with Candidiasis and hyposalivation. In addition, there was a greater frequency of frailty and candidiasis in women. The most common clinical forms of candidiasis are chronic hyperplastic and acute erythematous, with a higher prevalence of erythematous in females. Both types of candidiasis are strongly related to the use of dental prostheses. Longitudinal studies are recommended to evaluate the contributions of candidiasis and hyposalivation to the development and progression of fragility.

## Figures and Tables

**Table 1:** Associations between frailty, systemic diseases, oral status, and candidiasis.

Variable	Robust *n* (%)	Prefrailty *n* (%)	Frailty *n* (%)	Total	X^2^	*p* Value
**Systemic disease**					7.898	0.444
None	19 (6.4%)	197 (66.6%)	80 (27.0%)	296		
Arterial hypertension	4 (2.9%)	93 (66.9%)	42 (30.2%)	139		
Diabetes	2 (3.5%)	38 (66.7%)	17 (29.8%)	57		
AHT and diabetes	0 (0.0%)	17 (60.7%)	11 (39.3%)	28		
Other	1 (1.4%)	48 (69.6%)	20 (29.0%)	69		
**Smoking**					1.374	0.503
No	20 (4.4%)	309 (67.9%)	126 (27.7%)	455		
Yes	6 (4.5%)	84 (62.7%)	44 (32.8%)	134		
**Alcohol drinker**					3.937	0.140
No	15 (3.8%)	277 (69.3%)	108 (27.0%)	400		
Yes	11 (5.8%)	116 (61.4%)	62 (32.8%)	189		
**Use of prostheses**					0.409	0.815
No	9 (4.6%)	133 (68.2%)	53 (27.2%)	195		
Yes	17 (4.3%)	260 (66.0%)	117 (29.7%)	394		
**Subprosthetic stomatitis**					2.620	0.270
No clinical signs	10 (4.60%)	148 (68.2%)	59 (27.2%)	217		
Culture negative	5 (6.8%)	43 (58.1%)	26 (35.1%)	74		
Culture positive	2 (1.9%)	69 (67.0%)	32 (31.1%)	103		
**Mycological** **study** *					5.885	0.208
Not carried out	18 (5.0%)	245 (68.2%)	96 (26.7%)	359		
Positive	3 (2.5%)	85 (69.7%)	34 (27.9%)	122		
Negative	5 (4.6%)	63 (58.3%)	40 (37.0%)	108		
**Sialometry**					3.706	0.157
Normal	13 (4.2%)	217 (70.2%)	79 (25.6%)	309		
Hyposalivation	13 (4.6%)	176 (62.9%)	91 (32.5%)	280		

X^2^: chi-square test; ATH: arterial hypertension; * KOH (potassium hydroxide) and cultivation.

**Table 2:** Associations between *Candida* species types and location.

	Pseudomembranous Candidiasis *n* (%)	Erythematous Candidiasis *n* (%)	Atrophic Candidiasis *n* (%)	Hyperplastic Candidiasis *n* (%)	Total	Fisher	*p* Value
**Location**						103.731	0.000
Palate	4 (10.5%)	33 (86.8%)	1 (2.6%)	0 (0.0%)	38		
Tongue	15 (18.1%)	3 (3.6%)	8 (9.6%)	57 (68.7%)	83		
Buccal Mucosa	0 (0.0%)	0 (0.0%)	1 (100.0%)	0 (0.0%)	1		
**Total**	19 (15.6%)	36 (29.5%)	10 (8.2%)	57 (46.7%)	122		

**Table 3:** Associations between types of candidiasis and sociodemographic characteristics.

	Pseudomembranous Candidiasis *n* (%)	Erythematous Candidiasis *n* (%)	Atrophic Candidiasis *n* (%)	Hyperplastic Candidiasis *n* (%)	Total	X^2^	*p* Value
**Participants**	19 (15.6%)	36 (29.5%)	10 (8.2%)	57 (46.7%)	122		
**Gender**						11.991	0.006
Female	8 (9.9%)	31 (38.3%)	7 (8.6%)	35 (43.2%)	81		
Male	11 (26.8%)	5 (12.2%)	3 (7.3%)	22 (53.7%)	41		
**Level of education**						6.682	0.677
Incomplete learning	3 (14.3%)	5 (23.8%)	2 (9.5%)	11 (52.4%)	21		
Full primary	5 (10.2%)	17 (34.7%)	5 (10.2%)	22 (44.9%)	49		
High school completed	5 (15.6%)	11 (34.4%)	2 (6.3%)	14 (43.8%)	32		
Third level	6 (30.0%)	3 (15.0%)	1 (5.0%)	10 (50.0%)	20		
**Systemic diseases**						8.283	0.753
None	12 (20.7%)	13 (22.4%)	5 (8.6%)	28 (48.3%)	58		
AHT	3 (9.7%)	11 (35.5%)	4 (12.9%)	13 (41.9%)	31		
Diabetes	1 (9.1%)	4 (36.4%)	0 (0.0%)	6 (54.5%)	11		
AHT and diabetes	2 (20.0%)	5 (50.0%)	0 (0.0%)	3 (30.0%)	10		
Other *	1 (8.3%)	3 (25.0%)	1 (8.3%)	7 (58.3%)	12		
**Smoker**						3.468	0.323
No	13 (13.1%)	28 (28.3%)	9 (9.1%)	49 (49.5%)	99		
Yes	6 (26.1%)	8 (34.8%)	1 (4.3%)	8 (34.8%)	23		
**Consumes** **alcohol**						4.922	0.174
No	10 (11.4%)	27 (30.7%)	9 (10.2%)	42 (47.7%)	88		
Yes	9 (26.5%)	9 (26.5%)	1 (2.9%)	15 (44.1%)	34		
**Use of** **prostheses**						14.450	0.001
No	6 (31.6%)	0 (0.0%)	3 (15.8%)	10 (52.6%)	19		
Yes	13 (12.6%)	36 (35.0%)	7 (6.8%)	47 (45.6%)	103		

X^2^: chi-square test; AHT: arterial hypertension; * cardiac diseases, osteoarticular diseases, (mainly osteoporosis), etc.

**Table 4:** Relationships among fragility, subprosthetic stomatitis, and hyposalivation.

Subprosthetic Stomatitis Associated with Candidiasis			Robust *n* (%)	Prefrailty *n* (%)	Frailty *n* (%)	Total	Fisher’s Exact Test
	Sialometry *	Normal	2 (3.9%)	40 (78.4%)	9 (17.6%)	51	0.004
		Hyposalivation	0 (0.0%)	69 (67.0%)	32 (31.1%)	61	
**Total**			2	69	32	103	

* Unstimulated salivary flow.

## Data Availability

The data presented in this study are available on request from the corresponding author, as they are not publicly available due to ethical and privacy restrictions.
